# Enhancement of Aqueous Solubility and Dissolution of Celecoxib through Phosphatidylcholine-Based Dispersion Systems Solidified with Adsorbent Carriers

**DOI:** 10.3390/pharmaceutics11010001

**Published:** 2018-12-20

**Authors:** Kanghee Jo, Jae Min Cho, Hyunjoo Lee, Eun Kyung Kim, Hong Chul Kim, Hyeongmin Kim, Jaehwi Lee

**Affiliations:** 1 College of Pharmacy, Chung-Ang University, Seoul 06974, Korea; rkdgml2374@naver.com (K.J.); etecoli@hanmail.net (J.M.C.); hyunjoo1216@nate.com (H.L.); hm.kim8905@gmail.com (H.K.); 2 Graduate School of Pharmaceutical Management, Chung-Ang University, Seoul 06974, Korea; nu3eun@naver.com (E.K.K.); philipkhc@naver.com (H.C.K.)

**Keywords:** celecoxib, phosphatidylcholine, solid dispersion, solubility, dissolution rate

## Abstract

This study aimed to design phosphatidylcholine (PC)-based solid dispersion (SD) systems for enhancing the apparent aqueous solubility and dissolution of celecoxib (CLC), a selective cyclooxygenase-2 inhibitor with a highly hydrophobic property. Although PC-based dispersion formulations considerably increased solubilities of CLC, the lipidic texture of PC was not appropriate as a solid dosage form for oral administration of CLC. To mask the lipidic texture of PC-based matrices, Neusilin^®^ US2, an adsorbent material with a porous structure and large surface area widely used in the pharmaceutical industry, was employed and thereby fully powderized PC-based dispersion formulations could be fabricated. However, PC matrices containing CLC strongly adsorbed to the pores of Neusilin^®^ US2 was not able to be rapidly released. To address this problem, different hydrophilic materials were examined to promote the release of the CLC-dispersed PC matrices from Neusilin^®^ US2. Among tested hydrophilic materials, croscarmellose sodium was the most suitable to facilitate fast drug dissolution from Neusilin^®^ US2 particles, showing significantly enhanced apparent aqueous solubility and dissolution behavior of CLC. Through differential scanning calorimetry, X-ray diffraction, and Fourier transform infrared spectroscopy (FT-IR) analysis, a considerably reduced crystallinity of CLC dispersed in the PC-based dispersion formulations was demonstrated. The PC-based SD formulations developed in this study would be useful for improving the oral bioavailability of poorly soluble drugs such as CLC.

## 1. Introduction

Celecoxib (CLC) is one of the most commonly prescribed selective cyclooxygenase (COX)-2 inhibitors used for the treatment of acute and chronic pain and inflammation. Compared to non-selective non-steroidal anti-inflammatory drugs (NSAIDs) that inhibit COX-1 and COX-2, COX-2 inhibitors such as CLC have been known to cause fewer side effects such as upper gastrointestinal (GI) ulceration and bleeding [1,2,3]. CLC is classified as Class II drug in accordance with the Biopharmaceutics Classification System (BCS) and thus the oral bioavailability of CLC is largely governed by its low aqueous solubility of 1.15 µg/mL as measured in our laboratory and slow dissolution rate [4,5,6]. Therefore, it is necessary to increase the solubility and dissolution rate of CLC for the patients to achieve immediate pain relief after oral administration.

Many pharmaceutical scientists have attempted to improve the oral bioavailability of poorly soluble drugs using various pharmaceutical formulations such as liposomes, mixed micelles, and micro-/nano-emulsions as well as solid dispersion (SD) systems [7,8,9]. Among the various formulations described above, SD systems refer to a technique that produces the homogeneous molecular dispersion of hydrophobic drugs in an inert polymeric carrier to alter the crystalline state of the drugs to an amorphous state, which facilitates the fast dissolution of the drugs [5,6,10,11,12,13,14,15,16]. 

The majority of studies on SDs have generally used several hydrophilic polymers to prepare solid matrices in which hydrophobic drugs are molecularly dispersed in an amorphous state. The hydrophilic polymers not only carry the dispersed drugs but also assist the formation of an amorphous state of the drugs, leading to an increase in the absorption of the drugs [17,18]. Phospholipids are known to have an amphiphilic property and thereby are expected to show solubilizing potentials for hydrophobic drugs such as CLC [19,20]. However, owing to the lipidic texture of phospholipids, the preparation of solid dispersion systems in a fully powderized state using phospholipids is highly difficult [21,22,23]. 

In order to address this issue, we hypothesized that phospholipids-based dispersion systems of poorly soluble drugs could be powderized using inert adsorbent materials that have been used in the recent times for the purpose of preparing novel solid oral dosage forms in the pharmaceutical industry [9,24]. Various adsorbent carrier materials with different porous structures and adsorption capacities are currently available, such as Fujicalin^®^, Neusilin^®^, Sylysia^®^, and Aerosil^®^, and among them Neusilin^®^ US2 has been known to have a greater adsorption capacity than other adsorbent carriers because of its high porosity and large specific surface area [9,25]. Therefore, in the present study solid state phosphatidylcholine (PC)-based dispersion systems of CLC were designed using Neusilin^®^ US2 as a powderizing agent to enhance the apparent aqueous solubility and the dissolution behaviors of CLC in water for oral administration.

## 2. Materials and Methods 

### 2.1. Materials

CLC was kindly gifted from Dong Kwang Pharmaceutical Co. Ltd. (Seoul, Korea). Commercial CLC 200 mg tablet with the proprietary name of Celcok was obtained from Whanin Pharmaceutical Company (Seoul, Korea). Neusilin^®^ US2 (magnesium alumino metasilicate) was provided by Wooshin Medics Co. Ltd. (Seoul, Korea). PC was purchased from Lipoid GmbH (Ludwigshafen, Germany). Sodium starch glycolate (SSG), lactose, and croscarmellose sodium (CCS) were provided by WonPoong Pharmaceutical Company (Seoul, Korea). Polyvinylpyrrolidone (PVP) was obtained from BASF (Cleveland, OH, USA). Mannitol and dextrose were purchased from Daejung Chemicals & Metal Co., Ltd. (Siheung, Korea). Methanol and ethanol of high performance liquid chromatography (HPLC) grade were purchased from Honeywell-Burdick & Jackson (Muskegon, MI, USA).

### 2.2. Preparation of Phosphatidylcholine (PC)-Based Solid Dispersions (SDs)

The SDs of CLC were prepared with PC, Neusilin^®^ US2, different hydrophilic diluents and disintegrants by the solvent evaporation method. The compositions of the formulations are presented in Table 1. As for F1–F5, CLC and PC were added to ethanol and subsequently dispersed by stirring with a magnetic stirrer. The mixtures were transferred to a plate and the solvent was rapidly evaporated using a hair drier in a fume hood. In case of F6–F16, Neusilin^®^ US2 and/or the hydrophilic diluents and disintegrants were added to the mixtures of CLC and PC in ethanol and homogeneously mixed. The mixtures were then evaporated using the same method described above. The dried mixtures were then passed through a 100 µm sieve.

### 2.3. Evaluation of Apparent Aqueous Solubility of Celecoxib (CLC) Incorporated in PC-Based Dispersion Formulations 

An excess amount of pure CLC powders and PC-based dispersion formulations prepared were added to distilled water (2 mL) in screw-capped glass vials, and the formulations suspended in the vials were agitated using a magnetic stirrer at room temperature (~24 °C). After 12 hours, the suspensions were centrifuged (GYROGEN Co., Ltd., 1580 MGR, Daejeon, Korea) at 5,000 rpm for 10 min, and 100 µL of the supernatants were then diluted with 900 µL of methanol. The solutions were filtered using a 0.45 μm polyvinylidene fluoride syringe filter, and the levels of CLC in the solutions were evaluated using a high-performance liquid chromatography (HPLC) system as described below in detail.

### 2.4. High-Performance Liquid Chromatography (HPLC) Analysis of CLC

Levels of CLC in samples were assessed using HPLC (YL-9100, Young Lin Instrument, Co. Ltd., Anyang, Korea) based on a validated analysis method that was previously reported by Baboota et al. [26]. Hypersil GOLD™ C_18_ column (250 × 4.6 mm, 5 µm particle size, Thermo Fisher Scientific Inc., Waltham, MA, USA) was used. The mobile phase composed of methanol and water at a volume ratio of 75:25 flowed at a rate of 1.25 mL/min. The UV detection wavelength was set at 250 nm. 

### 2.5. Wicking Test of Hydrophilic Diluents and Disintegrants as a Water Absorption Enhancer 

Capillary glass tubes (internal diameter 1.55 mm × 75 mm height) were filled with selected hydrophilic diluents and disintegrants used for the preparation of PC-based SD formulations up to a height of 5 cm. The bottom ends of the capillary tubes were plugged with filter paper to prevent the leakage of the samples to the surrounding media. The bottom ends of the capillary tubes were slightly immersed in an aqueous solution of methylene blue to allow the solution to be absorbed by the disintegrants and diluents via capillary action. The time required for the solution to reach a height of 5 cm was measured for each sample.

### 2.6. Assessment of Flowability of PC-Based Dispersion Formulations Powderized with Neusilin^®^ US2

The flowability of PC-based dispersion formulations powderized with Neusilin^®^ US2 was evaluated by measuring the angle of repose and the flow rate through a funnel of the formulations. For the measurement of the angle of repose, a fixed funnel method was used [27,28]. A funnel with an orifice of an inner diameter of 10 mm specially designed for evaluating the flowability of powders or granules (Copley Scientific Ltd., Nottingham, United Kingdom) was fixed above a flat horizontal surface at an appropriate height. The orifice of the funnel was closed and the powderized PC-based dispersion formulations (F6–F16) of 10 g were poured into the funnel. After the orifice of the funnel was opened and the powderized PC-based dispersion formulations passed through the funnel, the angle of repose (θ) of the conical piles formed by the formulations was evaluated by calculating tan θ.

### 2.7. In Vitro Drug Dissolution Study

An in vitro drug dissolution study was performed using the USP dissolution Apparatus II. Pure CLC, PC-based dispersion formulations of F1–F16 equivalent to 30 mg of CLC, and commercial CLC 200 mg tablet were added to 900 mL of distilled water, and they were agitated at a paddle rotation speed of 100 rpm at 37 °C. The dissolution media of 2 mL were withdrawn at predetermined time points of 0.1, 0.2, 0.5, 1, 2, 4, 6, 8, 10, and 12 h from the vessels and the initial volume of the media was maintained by adding 2 mL of the fresh dissolution medium to each of the vessels. Levels taken of CLC in the dissolution media were evaluated using HPLC after filtering them through a 0.45 μm polyvinylidene fluoride syringe filter as described above.

### 2.8. Differential Scanning Calorimetry (DSC)

The samples were subjected to analyses with DSC, which was performed using an STA S-1000 differential scanning calorimeter (Linseis Inc., Robbinsville, NJ, USA). Pure CLC, PC, Neusilin^®^ US2, CCS, and F11 (5 mg) was transferred to an aluminum pan, respectively, and the temperature was increased from 30 to 350 °C at a heating rate of 10 °C/min.

### 2.9. X-Ray Powder Diffraction (XRD)

The X-ray powder diffraction (XRD) spectra of pure CLC, PC, Neusilin^®^ US2, CCS, and F11 were obtained by using an X-ray powder diffraction spectrometer (D8 Advance, Bruker AXS GmbH, Karlsruhe, Germany). The XRD data were collected at a scan speed of 6 deg/min, within a scan range of 3–40° of 2θ angles using a step size of 0.02. 

### 2.10. Fourier Transform Infrared (FT-IR) Spectroscopy

The FT-IR spectra of pure CLC and PC-based dispersion formulations (F5, F10, and F11) of CLC were obtained using an MB-100 BOMEM analyzer (Hartmann & Braun, Quebec, Canada). The samples (2 mg) were mixed with 200 mg of KBr and pressed into pellets. FT-IR spectra were obtained in a frequency range from 500 cm^−1^ to 4000 cm^−1^ by scanning each sample at a resolution of 4 cm^−1^.

### 2.11. Statistical Analysis

All experiments were conducted in triplicate. Means were compared by one-way analysis of variance and Student’s t-test. *P* < 0.05 was considered significant.

## 3. Results and discussion

### 3.1. Apparent Aqueous Solubility of CLC Incorporated in PC-Based Dispersion Formulations

#### 3.1.1. The Effect of the Amount of PC in PC-Based Dispersion Formulations on the Apparent Aqueous Solubility of CLC

The influence of the amount of PC in PC-based dispersion formulations on the apparent solubility of CLC was examined. Numerous literature has been presented that CLC belongs to BCS Class II [4,29,30], indicating the oral bioavailability of the drug is primarily limited by its solubility. The aqueous solubility of CLC is reported as being ~3–7 µg/mL at pH 7 and 37 °C [31]. Thus, only 1.75 mg of CLC can be dissolved in 250 mL of water, far less than the typical dose of CLC (i.e., 100–200 mg). Few studies have attempted to evaluate the effect of the CLC solubility on the absolute bioavailability of the drug. Paulson et al. reported that the absolute bioavailability of CLC evaluated with a solution (64–88%) was higher than that assessed with a capsule (22–40%) [31]. The CLC solution was prepared with a mixture of polyethylene glycol 400 and saline (2:1, *v*/*v*). This clearly implies that enhancing the solubility of CLC can increase the oral bioavailability of CLC. Therefore, in this study, we aimed to increase the aqueous solubility of CLC using PC-based dispersion formulations.

The apparent aqueous solubility of CLC was evaluated through HPLC analysis. The HPLC analysis conditions of CLC were set based on literature in which the HPLC analysis method was developed and validated [26]. The HPLC method for the quantification of CLC was validated accordingly and it was confirmed that the HPLC method provided suitable specificity, linearity, precision, and accuracy. 

**Figure 1:**
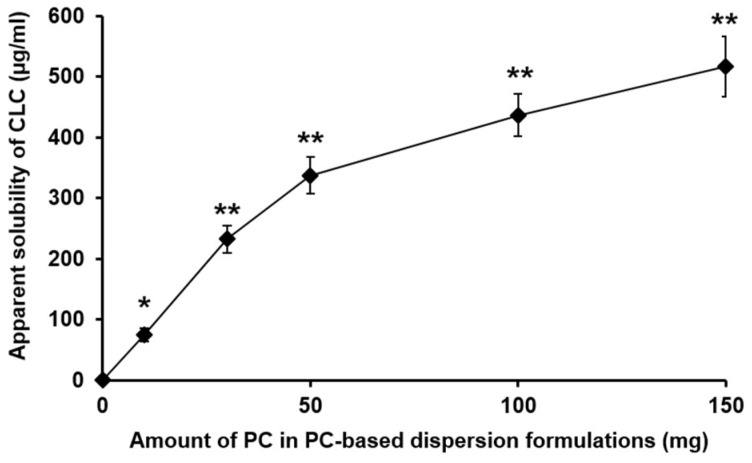
Apparent aqueous solubility of CLC incorporated in PC-based dispersion formulations prepared with different amounts of PC and the drug of 100 mg. The values are mean ± SD (*n* = 3). Single and double asterisks indicate the statistical differences at *P* < 0.001 and *P* < 0.0001, respectively, in comparison to the solubility of CLC alone.

As shown in Figure 1, the apparent aqueous solubility of CLC was considerably increased with increasing the amount of PC in the PC-based dispersion formulations tested. In particular, F5, a PC-based dispersion formulation containing the largest amount of PC among the tested formulations, exhibited approximately 450 times increased apparent aqueous solubility of CLC (516.67 µg/mL) compared with that of pure CLC (1.15 µg/mL). However, the dried PC-based CLC dispersions exhibited a lipidic texture, making it difficult to be formulated into an oral solid dosage form. Therefore, it was necessary to use an adsorption carrier to remove the lipidic texture of the PC-based CLC dispersions. 

#### 3.1.2. The Apparent Aqueous Solubility of CLC Evaluated from PC-Based Dispersion Formulations Powderized with the Adsorbent Carrier

PC-based dispersion systems using PC ranging from 10 mg to 200 mg were prepared to evaluate the aqueous solubility of CLC. The solubility study revealed that the aqueous solubility of CLC increased with an increasing amount of PC. Following the preparation of PC-based dispersion systems of CLC, we fabricated powderized PC dispersion systems of CLC with Neusilin^®^ US2, an adsorbent carrier material with a high adsorption capacity [32], to obtain solid state PC-based dispersion systems of CLC. However, due to the limited adsorption capacity of Neusilin^®^ US2, the PC-based dispersion systems having PC amounts exceeding 150 mg were not successfully powderized due largely to the lipidic texture of PC at room temperature. Thus, we prepared the PC-based dispersion systems powderized with Neusilin^®^ US2 up to the PC amount of 150 mg despite the fact that more PC could increase the aqueous solubility of CLC (Table 1).

**Figure 2:**
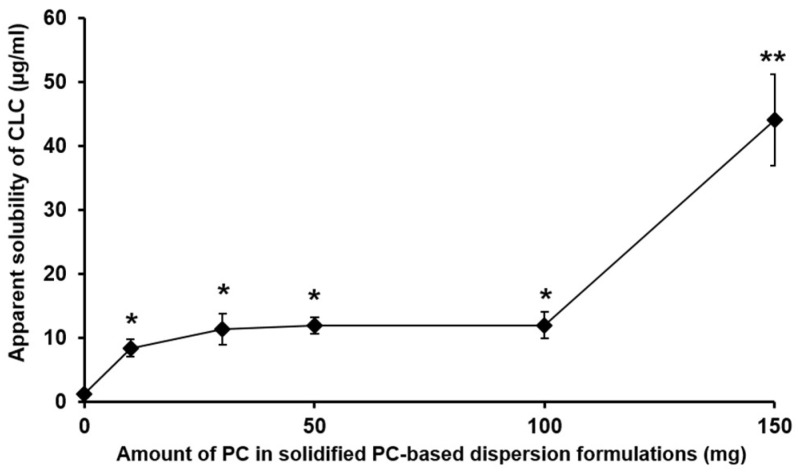
Apparent aqueous solubility of CLC incorporated in PC-based dispersion formulations powderized with Neusilin^®^ US2. The PC-based solid dispersion (SD) formulations tested were prepared with different amounts of PC, 100 mg CLC, and 200 mg Neusilin^®^ US2. The values are mean ± SD (*n* = 3). Single and double asterisks denote the statistical differences at *P* < 0.01 and *P* < 0.001, respectively, in comparison to the solubility of CLC alone.

Although the PC-based dispersion formulations were successfully powderized with Neusilin^®^ US2, the apparent drug solubility of the formulations was found to be significantly decreased (approximately 10–44 µg/mL) compared to those evaluated with PC-based dispersion formulations devoid of Neusilin^®^ US2 (F1–F5) (approximately 75–516 µg/mL) as depicted in Figure 2. The reason for this might be because the PC matrices containing CLC were adsorbed into the pores of Neusilin^®^ US2 and thereby the total amount of CLC was not able to be released from the absorbent carrier under the experimental condition [25]. We thus needed to use highly hydrophilic materials to facilitate the drug dissolution from the absorbent materials.

#### 3.1.3. Water Uptake Ability of Hydrophilic Diluents and Disintegrants

**Figure 3:**
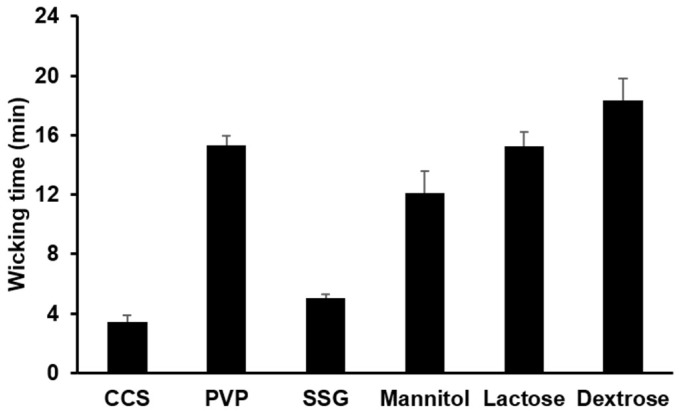
Water wicking time of different hydrophilic materials. The water wicking time was determined by measuring the time taken for the hydrophilic materials filled in capillary tubes at a height of 5 cm to fully absorb an aqueous solution of methylene blue. CCS, PVP, and SSG are abbreviations for croscarmellose sodium, polyvinylpyrrolidone, and sodium starch glycolate, respectively. The values are mean ± SD (*n* = 3).

Water uptake ability of different hydrophilic diluents and disintegrants that were used to prepare the PC-based SD formulations of F11–F16 was evaluated. The water uptake ability of the hydrophilic materials was examined by measuring the time taken for the materials filled in capillary tubes at a height of 5 cm to fully absorb an aqueous solution of methylene blue. As presented in Figure 3, the water wicking time of CCS was found to be the shortest, followed by SSG, mannitol, lactose, PVP, and dextrose. A shorter wicking time indicated a stronger water uptake ability of the hydrophilic materials that could be advantageous for promoting the penetration of water into the PC-based matrices adsorbed to Neusilin^®^ US2 and for facilitating the physical separation of the PC matrices from the adsorbent carriers.

#### 3.1.4. The Impact of Different Hydrophilic Diluents/Disintegrants as Water Absorption Enhancer in PC-Based Dispersion Formulations on the Apparent Aqueous Solubility of CLC

The solubility of CLC could significantly be enhanced by PC alone due largely to its amphiphilic nature and the amorphous state of the drug dispersed in the PC-based matrices. However, the PC-based CLC dispersions were difficult to handle as solid or powders due to the lipidic texture of PC, preventing the development of solid dosage forms. Thus, we attempted to powderize the PC-based CLC dispersions with mesoporous Neusilin^®^ US2. However, after the preparation of PC-based CLC dispersion powderized with Neusilin^®^ US2 we realized that the drug was difficult to be released and solubilized due to the adsorption of PC-based CLC dispersion systems into Neusilin^®^ US2. We, therefore, tried to add the hydrophilic materials such as hydrophilic diluents (mannitol, lactose, and dextrose) and disintegrants (CCS, PVP, and SSG) to facilitate the water uptake into spaces between PC-based CLC dispersion and Neusilin^®^ US2. This attempt successfully led to the promotion of physical separation of PC-based dispersion from Neusilin^®^ US2. The separated PC-based matrices were easily dispersed in water, thereby exhibiting the enhanced CLC solubilization and dissolution.

**Figure 4:**
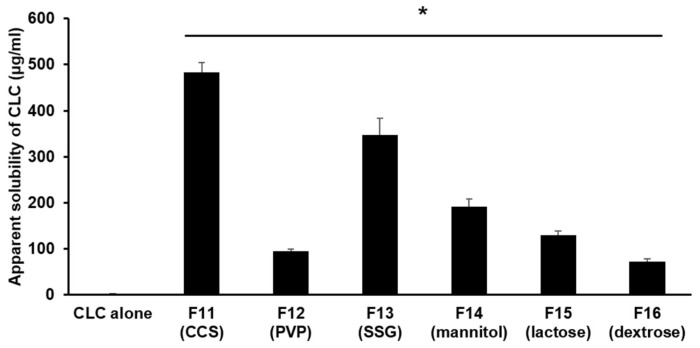
Apparent aqueous solubility of CLC incorporated in PC-based SD formulations containing different disintegrants (CCS, PVP, and SSG) and hydrophilic diluents (mannitol, lactose, and dextrose). The PC-based SD formulations tested were prepared with 150 mg PC, 100 mg CLC, 200 mg Neusilin^®^ US2, and 50 mg of each hydrophilic material (CCS, PVP, SSG, mannitol, lactose, and dextrose). CCS, PVP, and SSG are abbreviations for croscarmellose sodium, polyvinylpyrrolidone, and sodium starch glycolate, respectively. The values are mean ± SD (*n* = 3). The asterisk indicates the statistical difference at *P* < 0.0001 in comparison to the aqueous solubility of CLC alone.

**Figure 5:**
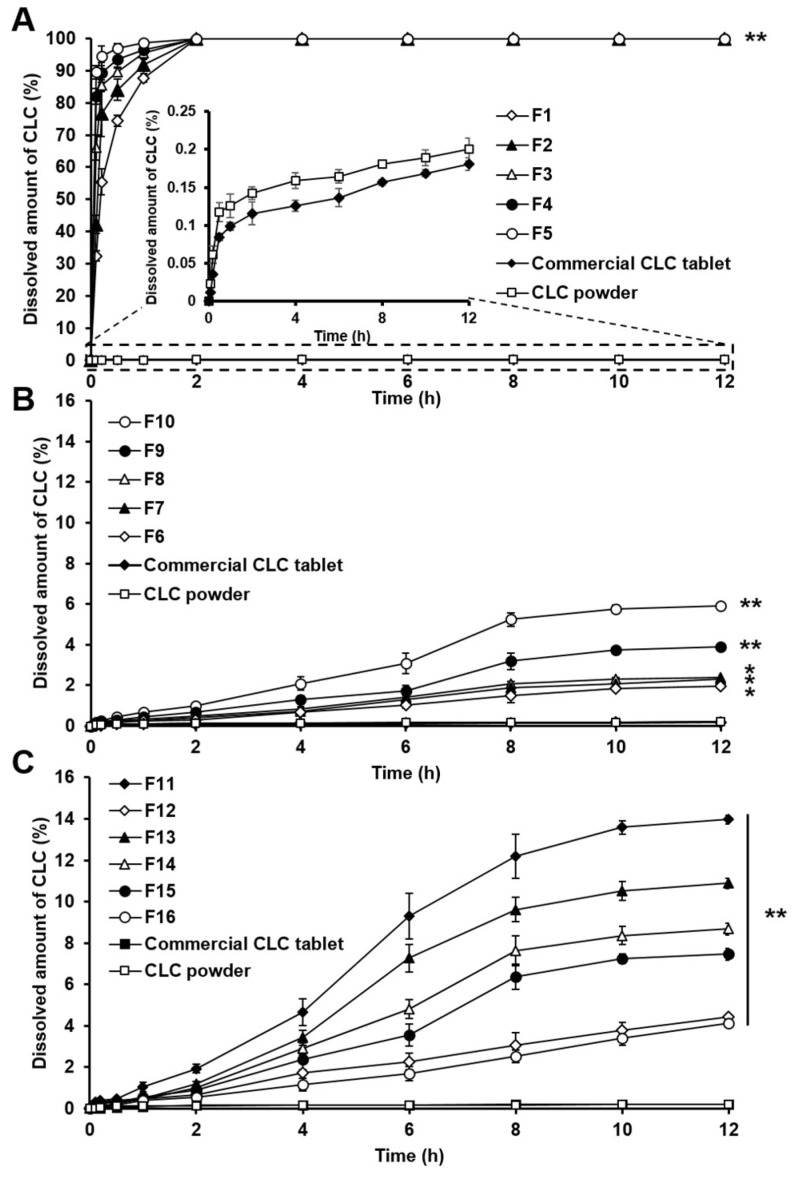
Dissolution profiles of CLC in water evaluated with (**A**) F1–F5, (**B**) F6–F10, and (**C**) F11–F16 for 12 h in comparison to those of commercial CLC 200 mg tablet and CLC powder. For clarity, the dissolution profiles of the commercial CLC tablet and CLC powder are expanded in the inserted graph in panel A. The dissolved amounts of CLC are presented as mean ± SD (*n* = 3) as a function of time up to 12 h. Single and double asterisks indicate the statistical differences in amounts of CLC dissolved at 12 h assessed with different PC-based dispersion formulations at *P* < 0.001 and *P* < 0.0001, respectively, compared to that observed with the commercial CLC tablet. In panel A, all dissolved amounts of CLC were statistically greater than those of the commercial CLC tablet and CLC powder at *P* < 0.0001 marked with double asterisks.

Figure 4 shows the result of the apparent aqueous solubility testing of PC-based SD formulations containing the hydrophilic water uptake enhancers such as disintegrants and hydrophilic diluents. The apparent aqueous solubilities of CLC evaluated with PC-based SD formulations containing CCS, PVP, SSG, mannitol, lactose, and dextrose were measured to be 483.33 ± 20.54, 95.00 ± 4.08, 346.66 ± 36.82, 191.67 ± 16.50, 130.01 ± 8.16, and 71.67 ± 6.24 µg/mL, respectively. In general, the apparent aqueous solubility of CLC examined with PC-based SD formulations containing disintegrants and hydrophilic diluents (F11–F16) was considerably increased compared to those assessed with PC-based SD formulations devoid of the hydrophilic materials (F6–F10). In particular, the solubility of CLC incorporated in powderized PC-based dispersions containing CCS was measured to be 483.33 µg/mL close to the highest solubility value (516.67 µg/mL) increased by PC alone matrices (F5). The aqueous solubility of CLC in water examined with F11 was also approximately 420 times greater than that of the CLC powder. The disintegrants and diluents were supposed to efficiently lead to the increased separation of PC-based CLC dispersions from the powderizing carriers at different degrees, which might be supported by different water absorbing capacity of each hydrophilic material shown in Figure 3 [27,28,29]. The increased solubilities of CLC also clearly caused the faster dissolutions of CLC as illustrated in Figure 5. Thus, it can be concluded that the hydrophilic materials with higher water uptake ability, especially CCS, were necessary to maintain the aqueous solubility of CLC greatly increased by PC-based dispersion systems through ameliorating the adsorption of the PC-based dispersions into Neusilin^®^ US2.

### 3.2. Flowability of PC-Based Dispersion Formulations Powderized with Neusilin^®^ US2

The flowability of PC-based dispersion formulations powderized with Neusilin^®^ US2 was evaluated by measuring the angle of repose and flow rate after pouring the formulations into a funnel with an orifice with an inner diameter of 10 mm. In case of the formulations devoid of Neusilin^®^ US2 (F1–F5), the flowability could not be assessed because of their lipidic texture caused by PC. As shown in Table 2, all the powderized PC-based dispersion formulations exhibited the angle of repose values (θ) ranging from 31 to 35. Based on the criteria for the evaluation of flowability of powders or granules provided by US Pharmacopeia, the flowability of the formulations was considered to be “good” [33]. The flow rates of the PC-based dispersion formulations were measured to be 4–6 g/s, which also implies that that the formulations were able to pass through the orifice of the funnel easily. Thus, it was demonstrated that the PC-based dispersion formulations could be successfully powderized by Neusilin^®^ US2 and thereby exhibited suitable flow properties to be formulated into solid dosage forms such as tablets and capsules. 

### 3.3. In Vitro Dissolution Study

The in vitro drug dissolution behaviors of pure CLC powders and PC-based dispersion formulations prepared were evaluated. It is known that the aqueous solubility of CLC with pK_a_ of 11.1 slightly increases with increasing pH of media [34]. For instance, the aqueous solubility of CLC has been reported to increase from 6 μg/mL to 11 μg/mL with increasing pH of media from 7.6 to 9.05 at 25 °C. Only under a strongly basic conditions (pH 10.9), the aqueous solubility of CLC considerably increases to 48 μg/mL. In this study, pH of the dissolution media was measured to be 6.5 before the start of the experiment, and it was slightly changed to 6.1–6.4 at the end of the experiment. The reason for the change in pH of the dissolution media might be because CLC is weakly acidic. On the basis of the aqueous solubility of CLC evaluated at different pH previously reported, the minor change in pH of the dissolution media might not significantly affect the dissolution behavior of CLC incorporated in the PC-based dispersion formulations.

As presented in Figure 5A–C, the CLC powder exhibited very low dissolved amounts of the drug with the maximum value of less than 1% at the end of the experiment. The dissolved amounts of CLC evaluated from the commercial tablet products were also very low (<1%), which was similar to those measured with CLC powder. This result might be because the dissolution experiment was performed in water as a dissolution medium where the solubility of CLC is very poor. According to US FDA, the dissolution of CLC formulations is evaluated with 0.04 M tribasic sodium phosphate and simulated gastric fluid containing 1% sodium lauryl sulfate [35]. The reason for this might be because of the fact that the dissolution experiment is performed as a means to evaluate the quality of the product and to monitor batch-to-batch differences. In this study, distilled water was used as a dissolution medium because our major effort was to increase the aqueous solubility and dissolution property of CLC using the amphiphilic phospholipid (PC)-based drug dispersion systems.

In case of the PC-based CLC dispersions devoid of Neusilin^®^ US2 (F1–F5), all of them showed significantly increased dissolved amounts of CLC for the whole tested period compared to those of the CLC powder and the commercial tablet products as shown in Figure 5A. In general, CLC of F1–F5 was completely dissolved within 2 h. Thus, it was demonstrated that the PC-based dispersions could enhance the drug dissolution rates considerably, which might be attributed to the amorphous state of CLC dispersed in the PC-based matrices. However, F1–F5 showed lipidic textures caused by PC that were not appropriate as oral solid dosage forms.

To powderize the PC-based dispersions, Neusilin^®^ US2 was added to the formulations as shown in Table 1 and the dissolution behaviors of CLC were examined with the PC-based dispersion systems powderized with Neusilin^®^ US2 (F6–F10). However, F6–F10 presented very low dissolved amounts of CLC during the tested period compared to those of F1–F5, showing the maximal dissolved drug amounts of approximately 2–6% at the end of the experiment as illustrated Figure 5B. The reason for this might be because the PC matrices incorporating CLC were adsorbed into Neusilin^®^ US2 and thereby the drug dissolution from the adsorbent carriers was considerably delayed. 

To facilitate the drug dissolution from the PC-based matrices powderized with Neusilin^®^ US2, different hydrophilic diluents and disintegrants were added to the PC-based SD formulations (F11–F16). As presented in Figure 5C, the PC-based SD formulations containing the hydrophilic materials showed appreciably increased drug dissolved amounts as a function of time compared to those of F6–F10, indicating that the hydrophilic materials promoted the drug dissolution successfully. In general, the formulation containing a hydrophilic material with a shorter water wicking time (Figure 3) exhibited greater drug dissolved amounts for the tested period (Figure 5C). This could be because the hydrophilic material with a stronger water uptake ability facilitated the penetration of the dissolution media into the PC-based matrices adsorbed to Neusilin^®^ US2 and thereby the PC-based matrices were separated from the adsorbent carriers. The separated PC-based matrices were supposed to be easily dissolved in the dissolution media, thereby exhibiting the solubilizing effect for CLC and enhancing the dissolution of the drug. Thus, it was demonstrated that the increased solubility of CLC caused by the powderized PC-based dispersion formulations containing the hydrophilic materials resulted in the improved dissolution behaviors of CLC.

### 3.4. DSC Study

**Figure 6:**
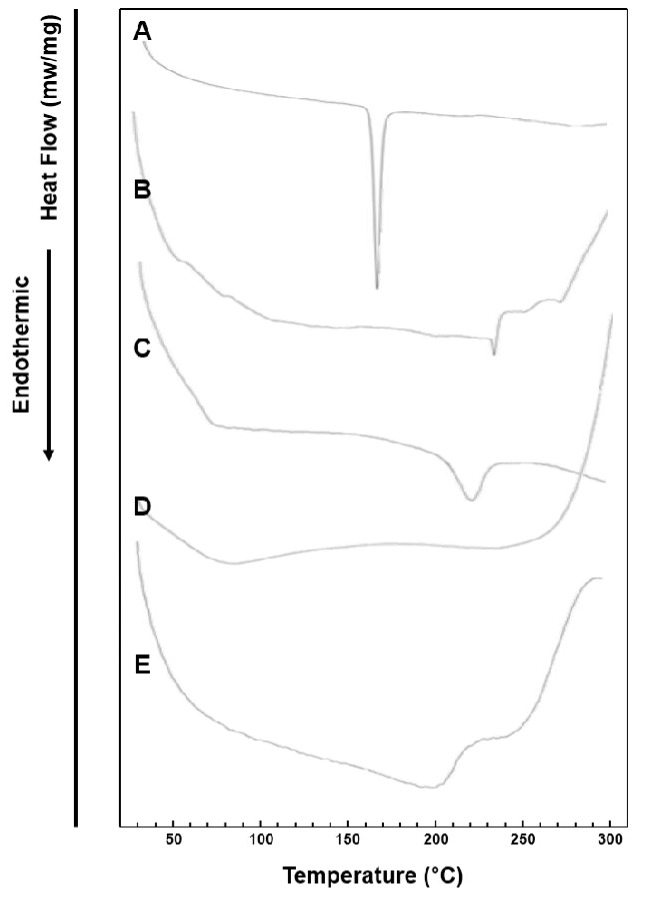
Differential scanning calorimetry (DSC) thermograms of (**A**) pure CLC, (**B**) PC, (**C**) Neusilin^®^ US2, (**D**) CCS, and (**E**) F11.

The DSC study was conducted to examine the melting profiles of CLC raw material powder, PC, Neusilin^®^ US2, CCS, the physical mixture of the ingredients, and the PC-based SD formulation (F11), which could be used to estimate their crystallinity [36]. Figure 6 shows the DSC thermograms of tested samples. The CLC powder (Figure 6A) presented a sharp endothermic peak at 163.19 °C that corresponded to the melting point of crystalline CLC [4,37]. In the thermogram of Neusilin^®^ US2 (Figure 6C) an endothermal peak was observed at a temperature of 219.06 °C, which was also in good agreement with previous studies [38,39]. In case of PC and CCS, they exhibited no endothermic peaks (Figure 6B,D). The characteristic peak of the crystalline CLC was not observed in the thermograms of F11) (Figure 6E), implying that CLC was successfully dispersed in the PC-based matrices in an amorphous state. When PC-based dispersion systems were prepared through the solvent evaporation method, PC and CLC were dissolved in ethanol and the ethanolic solutions were dried by warm air stream applied in a fume hood. As the evaporation procedure of ethanol progressed, PC was considered to form solid matrices and the drug molecules were supposed to be dispersed in the PC-based matrices in an amorphous state.

### 3.5. XRD Analysis

**Figure 7:**
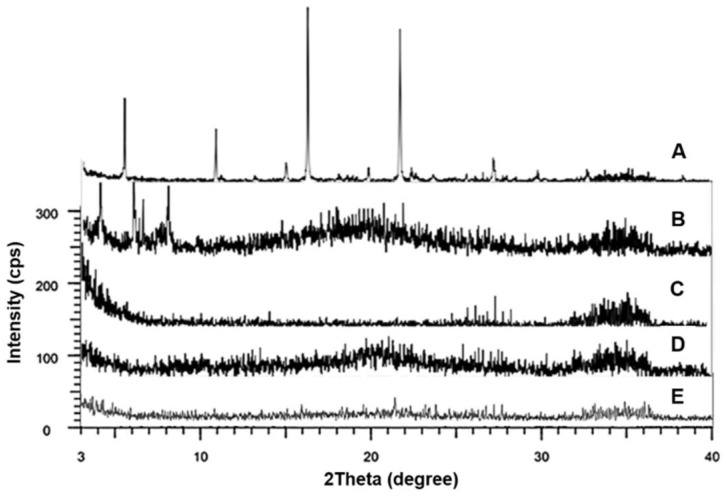
X-ray powder diffraction (XRD) patterns obtained from (**A**) pure CLC, (**B**) PC, (**C**) Neusilin^®^ US2, (**D**) CCS, and (**E**) F11.

XRD analysis was performed to confirm the reduced crystallinity of CLC dispersed in F11. Figure 7 depicts the X-ray diffraction patterns of CLC raw material powder, PC, Neusilin^®^ US2, CCS, and F11. The pure CLC exhibited characteristic XRD peaks at 2θ values of 5.3°, 10.7°, 16.1°, and 21.5° as previously reported [4,37,40]. PC also showed diagnostic peaks at 2θ values of 4.0°, 6.5°, 8.0°, and 14.7°. In case of the XRD patterns of F11, the intensity of the characteristic peaks shown by the CLC powder was significantly reduced, indicating that CLC was dispersed in the PC matrices in an amorphous state. This result was also in good agreement with the result of the DSC analysis, which also implied that the crystallinity of CLC was largely decreased.

### 3.6. FT-IR Spectroscopy

**Figure 8:**
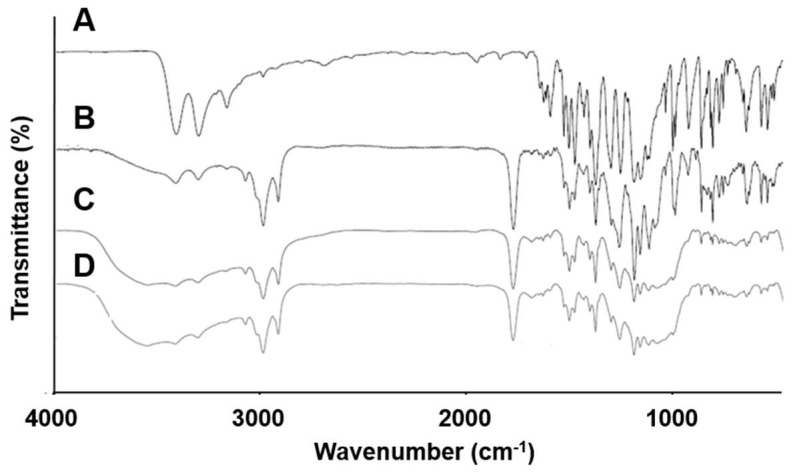
FT-IR spectra of (**A**) pure CLC, (**B**) F5 (CLC + PC), (**C**) F10 (CLC + PC + Neusilin^®^ US2), and (**D**) F11 (CLC + PC + Neusilin^®^ US2 + CCS).

The FT-IR spectra of pure CLC, F5 (CLC + PC), F10 (CLC + PC + Neusilin^®^ US2), and F11 (CLC + PC + Neusilin^®^ US2 + CCS) were obtained as illustrated in Figure 8. The FT-IR spectrum of CLC showed characteristic bands at 3338 and 3232 cm^−1^ (N–H stretching vibration of the SO_2_NH_2_ group), at 1347 and 1165 cm^−1^ (S=O asymmetric and symmetric stretching), and at 1230 cm^−1^ (C–F stretching) [4,37,40]. The spectrum of PC showed a band at 1253 cm^−1^ (P=O stretching). As for the PC-based dispersion formulations (F5, F10, and F11), the wavenumbers of peaks for the N–H stretching vibration and P=O stretching were considerably shifted as shown in Table 3. The reason for this might be owing to the hydrogen bonds between CLC and PC formed during the preparation procedure of the PC-based dispersion formulations. This result supported that CLC was well dispersed in PC-based matrices of the formulations and interacted with PC.

## 4. Conclusions

PC-based dispersion formulations for enhancing the apparent aqueous solubility and dissolution of CLC were developed. Although CLC molecularly dispersed in PC alone showed considerable increase in the aqueous solubility of CLC in water, PC-based CLC dispersion was not suitable for the development of solid dosage forms due to the lipidic texture of PC. Thus, PC-based CLC dispersion was powderized with Neusilin^®^ US2. The hydrophilic diluents and disintegrants were necessary to reproduce the increased aqueous solubility of CLC by PC-based dispersion systems. The water wicking ability correlated to increasing the aqueous solubility of CLC in PC-based dispersions adsorbed with Neusilin^®^ US2. In conclusion, the apparent aqueous solubility of CLC and its dissolution rates were significantly enhanced by the PC-based SD formulations largely owing to the amorphous state of CLC dispersed in the PC matrices, which was demonstrated by DSC, XRD, and FT-IR analysis. The PC-based SD formulations developed in this study would be useful for improving the oral bioavailability of poorly soluble drugs such as CLC.

## Figures and Tables

**Table 1:** Compositions of phosphatidylcholine (PC)-based dispersion formulations for celecoxib (CLC).

Formulation Code	Celecoxib (mg)	PC (mg)	Neusilin^®^ US2 (mg)	Disintegrants			Diluents		
				CCS (mg)	PVP (mg)	SSG (mg)	Mannitol (mg)	Lactose (mg)	Dextrose (mg)
F1	100	10	-	-	-	-	-	-	-
F2	100	30	-	-	-	-	-	-	-
F3	100	50	-	-	-	-	-	-	-
F4	100	100	-	-	-	-	-	-	-
F5	100	150	-	-	-	-	-	-	-
F6	100	10	200	-	-	-	-	-	-
F7	100	30	200	-	-	-	-	-	-
F8	100	50	200	-	-	-	-	-	-
F9	100	100	200	-	-	-	-	-	-
F10	100	150	200	-	-	-	-	-	-
F11	100	150	200	50	-	-	-	-	-
F12	100	150	200	-	50	-	-	-	-
F13	100	150	200	-	-	50	-	-	-
F14	100	150	200	-	-	-	50	-	-
F15	100	150	200	-	-	-	-	50	-
F16	100	150	200	-	-	-	-	-	50

**Table 2:** The angle of repose and flow rate of PC-based dispersion formulations powderized with Neusilin^®^ US2 measured after pouring the formulations into a funnel with an orifice of an inner diameter of 10 mm. The values are mean ± SD (*n* = 3).

Formulation Code	Angle of Repose (θ)	Flow Rate (g/s)
F6	31.15 ± 0.92	4.43 ± 0.22
F7	31.65 ± 0.37	4.50 ± 0.15
F8	32.01 ± 0.55	4.74 ± 0.19
F9	33.59 ± 0.71	4.90 ± 0.20
F10	34.94 ± 0.34	5.54 ± 0.28
F11	34.37 ± 0.35	5.76 ± 0.31
F12	34.93 ± 0.48	5.95 ± 0.30
F13	34.11 ± 0.70	5.43 ± 0.27
F14	34.08 ± 0.64	5.83 ± 0.34
F15	34.59 ± 0.84	5.44 ± 0.29
F16	34.25 ± 0.43	5.87 ± 0.13

**Table 3:** Wavenumber of peaks for S=O asymmetric and symmetric stretching and N–H stretching vibration of CLC and P=O stretching of PC evaluated from CLC raw material powder, F5 (CLC + PC), F10 (CLC + PC + Neusilin^®^ US2), and F11 (CLC + PC + Neusilin^®^ US2 + CCS).

	Wavenumber of Peaks (cm^−1^)		
	CLC		PC
	S=O (cm^−1^)	NH_2_ (cm^−1^)	P=O (cm^−1^)
CLC powder	1165/1347	3232/3338	1253
F5	1164/1347	3234/3339	1235
F10	1165/1348	3235/3343	1232
F11	1165/1348	3237/3349	1232
